# Small fish, big discoveries: zebrafish shed light on microbial biomarkers for neuro-immune-cardiovascular health

**DOI:** 10.3389/fphys.2023.1186645

**Published:** 2023-06-01

**Authors:** Hemaa Sree Kumar, Alexander S. Wisner, Jeanine M. Refsnider, Christopher J. Martyniuk, Jasenka Zubcevic

**Affiliations:** ^1^ Department of Physiology and Pharmacology, University of Toledo, Toledo, OH, United States; ^2^ Department of Neuroscience and Neurological Disorders, University of Toledo, Toledo, OH, United States; ^3^ Department of Medicinal and Biological Chemistry, University of Toledo, Toledo, OH, United States; ^4^ Center for Drug Design and Development, College of Pharmacy and Pharmaceutical Sciences, University of Toledo, Toledo, OH, United States; ^5^ Department of Environmental Sciences, University of Toledo, Toledo, OH, United States; ^6^ Department of Physiological Sciences, College of Veterinary Medicine, University of Florida, Gainesville, OH, United States

**Keywords:** gut microbiota, aquatic health, animal models, cardiovascular system (CVS), nervous system, immune system, zebrafish

## Abstract

Zebrafish (*Danio rerio*) have emerged as a powerful model to study the gut microbiome in the context of human conditions, including hypertension, cardiovascular disease, neurological disorders, and immune dysfunction. Here, we highlight zebrafish as a tool to bridge the gap in knowledge in linking the gut microbiome and physiological homeostasis of cardiovascular, neural, and immune systems, both independently and as an integrated axis. Drawing on zebrafish studies to date, we discuss challenges in microbiota transplant techniques and gnotobiotic husbandry practices. We present advantages and current limitations in zebrafish microbiome research and discuss the use of zebrafish in identification of microbial enterotypes in health and disease. We also highlight the versatility of zebrafish studies to further explore the function of human conditions relevant to gut dysbiosis and reveal novel therapeutic targets.

## Zebrafish as an aquatic model in microbiota research

The microbiome is composed of bacteria, archaea, fungi, viruses, and parasites, with genetic and functional differences (Cresci and Bawden, 2015) and existing in evolutionary homeostatic symbiosis with each other and the host. Human microbiome is first established at birth, and it continues to evolve throughout lifetime under the influence of endogenous (host) and environmental factors including diet, medications, evolutionary constraints (e.g., geographical location), and aging, among others (Cresci and Bawden, 2015). Owing to the development of next-generation DNA sequencing, the intricate relationship between a host and its resident microbial population, and particularly the gut bacteria, is now studied in greater detail (de Vos et al., 2022).

**FIGURE 1:**
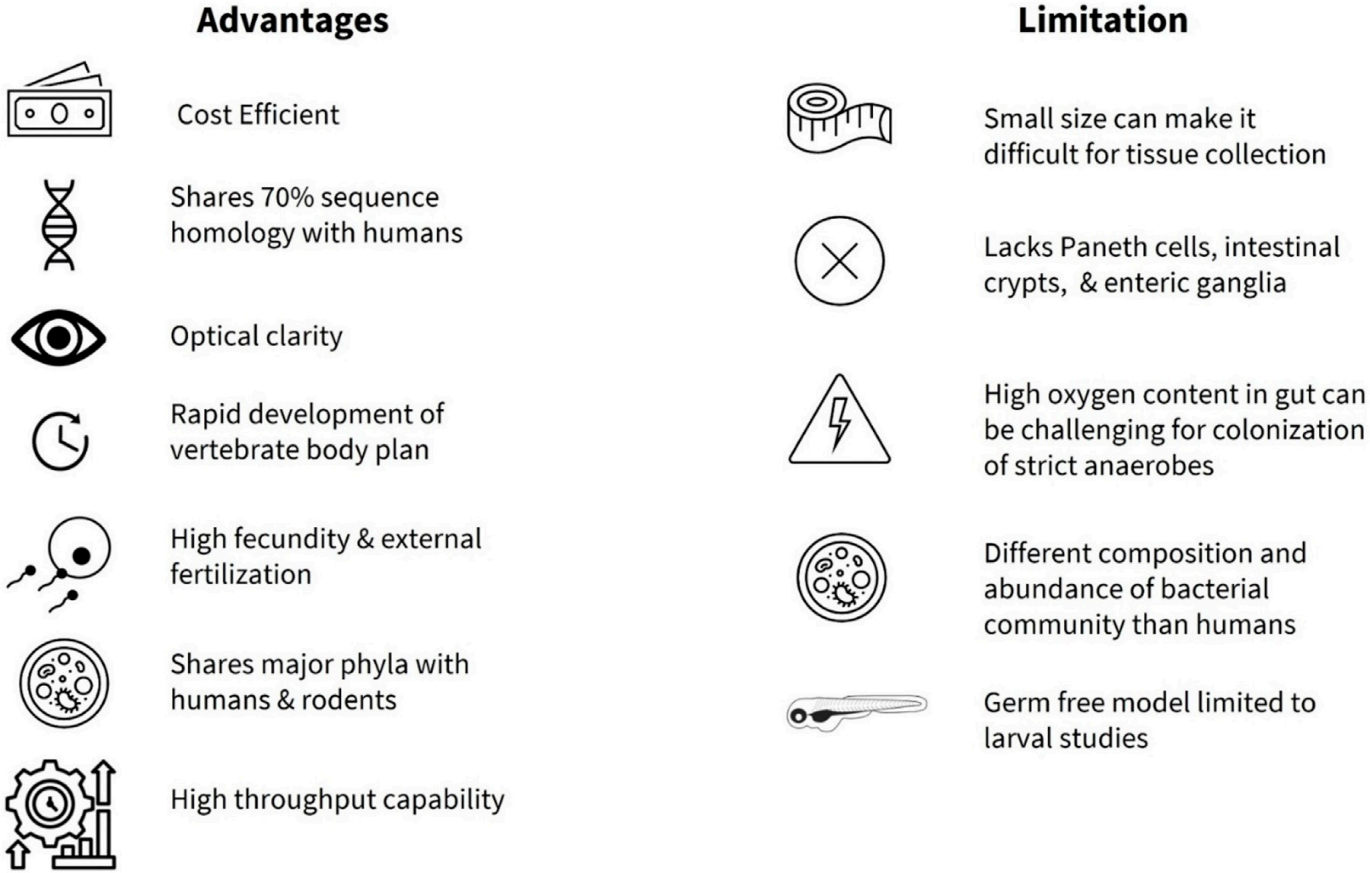
Summary of advantages and limitations of zebrafish as a model organism to study gut microbiota.

Zebrafish (*Danio rerio*), members of the *Cyprinidae* family (Reed and Jennings, 2011), are an increasingly popular vertebrate model organism in biomedical research. Advantages of the zebrafish as a model organism include ease and cost-effectiveness of animal husbandry, high fecundity, and high throughput capability (Reed and Jennings, 2011) (Figure 1). Zebrafish also share a high degree of sequence homology (∼70%) as well as conservation of function with many human proteins, with estimated 82% similarity of orthologs for human disease-related genes (Howe et al., 2013). Due to the availability of the whole genome sequence with more than 26,000 protein-coding genes (Howe et al., 2013), the zebrafish model offers a valuable reference to human disease. A particular advantage of zebrafish research is that, developmentally, all major organ systems including a beating heart, major vessels with circulating blood, and a rudimentary gut, are fully formed by 48 h post fertilization (hpf) (Gut et al., 2017). In addition, zebrafish are transparent in early developmental stages, while some transgenic lines such as Casper, a r*oy;nacre* double homozygous mutant, remain transparent throughout their lifetime (White et al., 2008) enabling *in vivo* imaging, high throughput chemical screening (Tackie-Yarboi et al., 2020) and drug discovery studies (MacRae and Peterson, 2015). Furthermore, genetic manipulation techniques such as morpholino (Nasevicius and Ekker, 2000), CRISPR-Cas9 system (Hwang et al., 2013; Albadri et al., 2017), Gal4/UAS system (Scheer and Campos-Ortega, 1999; Zhang et al., 2019a), and Cre/loxP system (Boniface et al., 2009) are now routinely used in zebrafish research of human disorders (Santoriello and Zon, 2012). The ease of gene manipulation allows screening of small molecules in digestive system development, innate immunity in the gut, dietary lipid metabolism, and gut motility (MacRae and Peterson, 2015).

Adopting zebrafish in basic research began with George Streisinger and colleagues at the University of Oregon in the late 1960s, who studied the development and function of the nervous system (Eisen Cartner et al., 2020). Since his seminal study (Streisinger et al., 1981), the zebrafish have been utilized to characterize molecular, cellular, and genetic mechanisms of developmental processes (Grunwald and Eisen, 2002), as well as a variety of neurologic, physiologic and metabolic processes and disorders (Choi et al., 2021). The zebrafish model is now utilized in many cases to study various human diseases ahead of rodents in cancer research (Ignatius et al., 2016; Hason and Bartůněk, 2019), drug discovery (Cassar et al., 2020), and toxicity studies (Horzmann and Freeman, 2018; Lai et al., 2021). More recently, zebrafish have been used in investigations of the gut microbiota related to nutrient metabolism, neural development, immune system homeostasis, and pathophysiologic processes that underlie human diseases (Diaz Heijtz et al., 2011; Roeselers et al., 2011; Clemente et al., 2012; Shreiner et al., 2015; de Abreu et al., 2019; Stagaman et al., 2020; de Vos et al., 2022; Zhong et al., 2022). One advantage of using zebrafish in study of host-microbiota interactions is that organ formation is apparent at 3 days post fertilization (dpf), allowing for external microbial colonization (Rawls et al., 2004; Bates et al., 2006a; Kanther and Rawls, 2010) from larval stages. Functionally, there is a great deal of overlap with the mammalian gut, with enteric nervous system (ENS), mucin-secreting goblet cells, absorptive enterocytes, hormone releasing enteroendocrine cells (EECs), immune cells, and smooth muscle cells, all present early on in development (Wallace and Pack, 2003; Wallace et al., 2005). However, some notable differences in the anatomy of the gastrointestinal (GI) tract are also present (Kuil et al., 2020). One limitation is that the zebrafish do not possess Paneth cells, intestinal crypts, or a stomach (Figure 1), and the intestine is connected directly to the esophagus (Ye et al., 2019). In addition, the zebrafish ENS is much simpler when compared to other vertebrates. For example, the ENS in zebrafish originates mainly from vagal neural crest and they lack submucosal ganglia and myenteric neurons compared to vertebrates (Shepherd and Eisen, 2011).

Another advantage of using the zebrafish is the evidence for a shared core gut microbiota (e.g., *Proteobacteria*, *Firmicutes*, *Bacteroidetes*, *Actinobacteria*, and *Verrucomicrobia*) between humans, rodents, and the zebrafish at the phylum level (Roeselers et al., 2011; Sweeney and Morton, 2013; de Abreu et al., 2019; Li et al., 2022; Xia et al., 2022) (Figure 1). As in mammals, the gut microbiota profile in the zebrafish shifts with age, with *Proteobacteria* being more abundant in the larval stage, while *Firmicutes* and *Fusobacteria* are more prevalent in the adult zebrafish gut (Kanther and Rawls, 2010; Xia et al., 2022). In addition, the zebrafish microbiota is relatively stable and shows conservation between bacterial taxa in the natural habitat and research facilities (Roeselers et al., 2011), while the gut microbiota of male and female zebrafish reportedly does not differ significantly in laboratory environments (Martyniuk et al., 2022). In an important paper, Stagman et al. reported that experimental methods influence how we interpret effects of xenobiotics or exposure of environmental factors on the gut microbiota, information that can guide researchers towards adequate design and execution of studies (Stagaman et al., 2022). Their findings demonstrated that abundance and composition of microbial biomarkers were significantly impacted by the dissection method (gut dissection vs. whole fish) and the DNA extraction kit, whereas the inclusion of PCR replicates had minimal effect (Stagaman et al., 2022). Importantly, zebrafish can be reared as germ-free with gnotobiotic husbandry practices (Rawls et al., 2004; Milligan-Myhre et al., 2011; Melancon et al., 2017; Jia et al., 2021) and colonized by commensal bacteria for direct investigation of host-microbiota interactions (Pham et al., 2008; Jia et al., 2021). In addition, utilization of multi-colonization system (e.g., conventional vs. axenic vs. colonized) as described in Catron et al., allows the use of larval zebrafish for high throughput screening in exploration of relationships between gut microbiota, xenobiotics and exposomes (Catron et al., 2019) in incidence of cardio-metabolic diseases and neuronal dysregulation (Daiber et al., 2019; Bertotto et al., 2020; Frenis et al., 2021). One of the limitations in germ-free studies is the restriction to using larval zebrafish, as the nutritional requirement and methodology to maintain germ-free zebrafish through adulthood are not fully understood (Kanther and Rawls, 2010; Xia et al., 2022) (Figure 1). However, a recent study by Zhang and colleagues addressed this in part by administering a cocktail of antibiotics to successfully deplete the host endogenous microbiome in the adult zebrafish (Zhang et al., 2023). Humanized zebrafish larvae have also shown success with transplantation of whole human intestinal microbiota (Arias-Jayo et al., 2018a; Valenzuela et al., 2018), while colonization with specific anaerobes from major human bacterial phyla (*Actinobacteria*, *Bacteroidetes*, *Firmicutes*) are achieved by static immersion and microinjection techniques (Toh et al., 2013). Long term colonization of adult zebrafish with live gut anaerobes or strictly anerobic bacteria currently presents a challenge, compounded by the elevated intestinal oxygen levels in the zebrafish (Lu et al., 2021) (Figure 1), although administration of lyophilized bacteria has been successful under certain conditions (Borrelli et al., 2016; Zhang et al., 2021a; Liu et al., 2023).

## Potential for zebrafish as a unifying tool to investigate the complex multisystem microbiota-host interactions

There is a growing understanding of the complexity of host-microbiota in development and overall host homeostasis (Afzaal et al., 2022). An imbalance in the gut microbiota, commonly termed gut dysbiosis, is functionally associated with many human conditions including anxiety and depression (Naseribafrouei et al., 2014; Carabotti et al., 2015), obesity (Lee et al., 2020), diabetes (Iatcu et al., 2021; Lau et al., 2021), inflammatory diseases (Glassner et al., 2020), hypertension (Yang et al., 2015; Naqvi et al., 2021) and other cardiovascular diseases (Afzaal et al., 2022). As an example, stimulation of the nervous system by chronic infusion of angiotensin II, a peptide with cardiovascular properties, can modulate the gut bacteria in rats (Yang et al., 2015; Santisteban et al., 2017; Donertas Ayaz et al., 2021), and recruit splenic immune cells in mice, demonstrating a complex host-microbiota multisystem interaction (Carnevale et al., 2020). In addition, transplant of gut microbiota from hypertensive rodents induces hypertension, elevate the sympathetic drive, and activate the immune system in rats (Toral et al., 2019a; Toral et al., 2019b). Thus, experimental evidence strongly supports an integrated gut-brain-immune axis that involves both microbial communities and the host, with a growing need for animal models to study these complex interactions.

**FIGURE 2:**
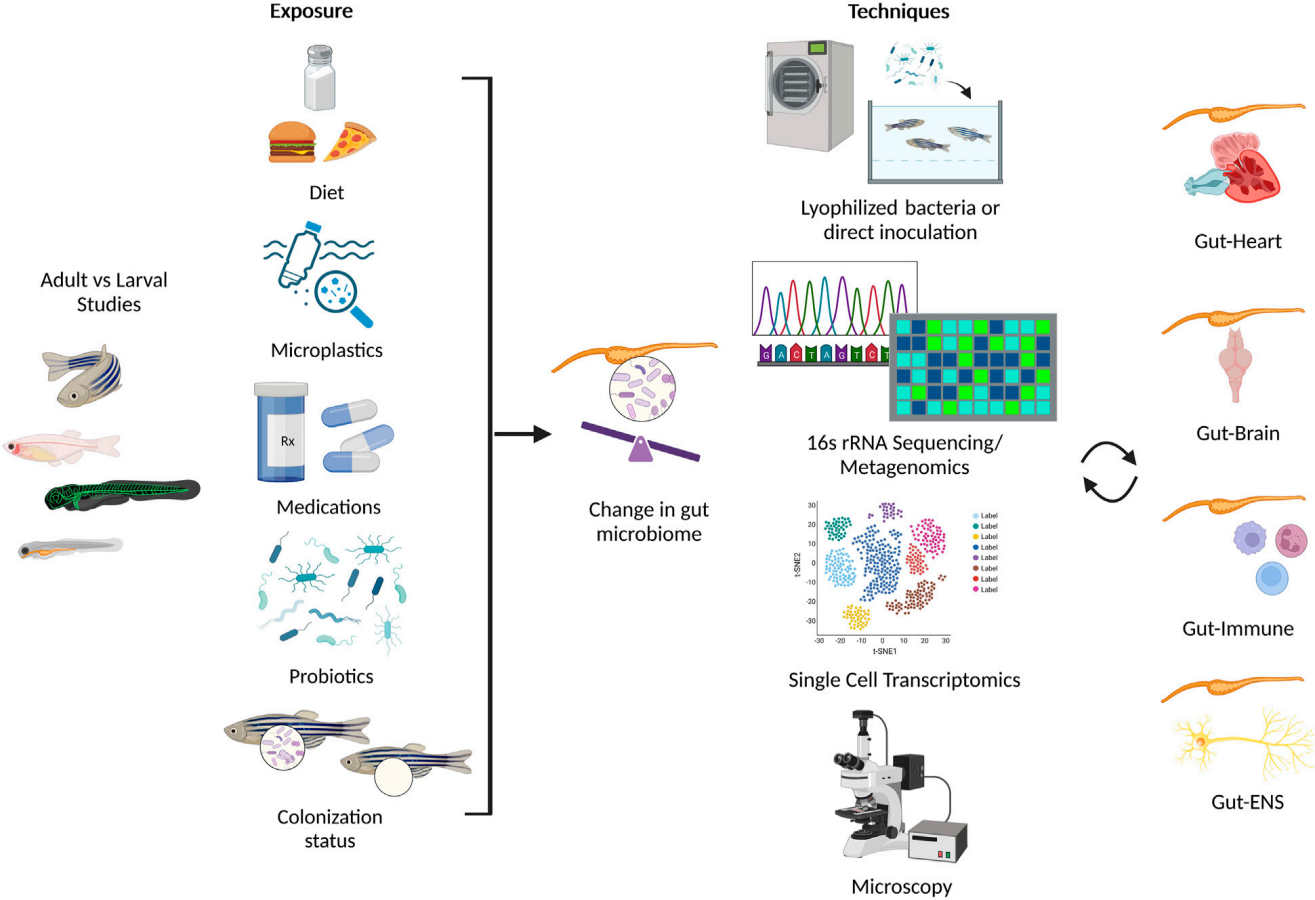
Summary of environmental conditions that alter the gut microbiome in zebrafish (left panel), and assorted techniques used to investigate the effect of gut microbiota on multiple systems in zebrafish (right panel). Created with BioRender.com.

Although there are a limited reports in zebrafish that investigate the cross-talk between multiple systems (Rolig et al., 2017; Zang et al., 2019; Ding et al., 2021; Nie et al., 2022; Tian et al., 2023; Zhang et al., 2023), their physiologic advantages show promise towards multisystem approaches (Jemielita et al., 2014). Recently, a statical technique known as the Latent Dirichlet Allocation examined the potential for advancement of research topics such as “Gut Microbiota” and “Metabolic Pathway” and recommended the focus on three topics in future studies: i) metabolomics-based approaches and gut microbial metabolites, ii) metabolic disease, and iii) brain function and cardiovascular disease (Ning et al., 2020). Here, we highlight the potential of zebrafish as a model organism to study the intricate relationship between the gut microbiota and the host neural, immune, and cardiovascular systems, as well as a potential biomarker in health and disease (Figure 2; Table 1).

**TABLE 1:** Summary of gut microbiota modulation in cardiovascular, neural, and immune system in zebrafish.

Model of zebrafish	Effector	Method of administration	Effects	References
Cardiovascular				
Larval zebrafish	High fat diet	Direct administration with feed in rearing water	Gene ontology enrichment analysis showed that genes associated with liver development, blood coagulation, triglyceride homeostasis and response to oxidative stress were altered, increased expression of genes associated with hyperlipidemia such as *cyp 51, acox1, hmox1a*, and *fads2* as well as increase in the activity of signaling pathways associated with metabolic regulation in zebrafish on high fat diet.	Ka et al. (2020)
Germ free larvae	Fecal microbiota transplant (FMT) from mouse on high fat diet	Immersion in sterile E3 embryo media	Larvae that received FMT was more susceptible to hyperlipidemia, which was driven by *Stenotrophomonas maltophilia* and *Enterococcus faecalis*, evidenced by the increase in lipid droplet accumulation, while inoculation of *E. faecalis* in zebrafish larvae with *myd88* knockdown showed significantly less lipid droplet accumulation.	Manuneedhi Cholan et al. (2022)
	*Enterococcus faecalis*			
Larval zebrafish	*Lactobacillus rhamnosus*	Direct inoculation in rearing water	Administration of *L. rhamnosus* increased the abundance of Firmicutes and reduced abundance of Actinobacteria, modulated lipid metabolism by decreasing expression of *fit2, agpat4, dgat2, mgll, hnf4α, scap, and cck* which is involved in metabolism of cholesterol and triglyceride, increased absorptive surface area, and improved the growth of the larvae compared to the control group.	Falcinelli et al. (2015)
Larval zebrafish	*Lactobacillus rhamnosus*	Direct inoculation in rearing water	Administration of *L. rhamnosus* increased the abundance of Firmicutes, reduced abundance of Actinobacteria, increased expression of *nucb2a*, *glp-1*, *insulin*, *goat, leptin*, decreased expression of *mc4r, cb1* and *npy,* which decreased glucose levels and appetite and smaller lipid droplets, higher levels of SCFA accumulated in the intestine and gallbladder compared to control group.	Falcinelli et al. (2016)
Germ free larvae	-	-	Decreased pro-angiogenic factors in the intestine and genes associated with regulation of leukocyte migration, cell proliferation and sprouting angiogenesis.	Willms et al. (2022)
Adult zebrafish	Overfeeding regime	Direct exposure in rearing water	Decrease in abundance of *Fusobacteria*, and *Tenericutes* and increase in *Proteobacteria.*	Buerger et al. (2020)
	*Diethylhexyl phthalate*			
Neural				
Adult zebrafish	Lyophilized *L. rhamnosus* IMC 501	Direct inoculation in rearing water	Increase in shoaling and explorative behavior, change in abundance of *Fusobacteria*, *Proteobacteria*, *Firmicutes*, *Cyanobacteria* at phylum level in treatment group, increase in expression of *bdnf*, *tph1a, tph1b*, *tph2*, *htr1aa*, *slc6a4a*, and *mao* in the brain, and decrease in expression of *tph1b*, *htr1aa* in the gut.	Borrelli et al. (2016)
Adult zebrafish	Enrofloxacin	Direct inoculation in rearing water	Increase in anxiety like behavior, increase in *Bacteroidetes*, decrease in *Firmicutes/Bacteroidetes* ratio, increase in intestinal levels of IL-6, TNF-α, 5-HT, and decrease in corticotropin-releasing hormone, BDNF, and neuropeptide Y in the brain.	Tian et al. (2023)
Larval zebrafish	*Vibrio*	Inoculation in flask	Zebrafish lacking *sox10* gene lacks a functional ENS resulted in intestinal bacterial overgrowth with increase in pro-inflammatory bacteria (*Vibrio*) and decrease in anti-inflammatory bacteria (*Escherichia*) as well as increased intestinal inflammation as evidenced by increase in intestinal neutrophils and increased epithelial cell proliferation which was dependent on TNF signaling. Colonization of mutant larvae with *Escherichia* and *Shewanella* as well as transplant of ENS precursors from wild-type donor rescued the inflammatory effects.	Rolig et al. (2017)
	*Shewanella*			
	*Escherichia*			
Larval zebrafish	*Edwardsiella tarda*	Immersion	Administration of *E. tarda* activated EEC via transient receptor potential ankyrin A1 (Trpa1), increased intestinal motility and activation of EECs stimulated the vagal sensory ganglia and cholinergic enteric neurons in the gut by releasing serotonin.	Ye et al. (2021)
Germ free larvae	*Lactobacillus plantarum*	Direct inoculation in rearing water	Germ free larvae showed increase in locomotor activity and in anxiety related behavior. Supplementation with *Lactobacillus plantarum* decreased anxiety like behavior. Change in relative abundance at phylum level with lower abundance of *Bacteroidetes*, *Cyanobacteria* and higher abundance of *Proteobacteria* in CV compared to CR larvae.	Davis et al. (2016a)
Conventionalized (CV) and conventionally raised (CR) larvae				
Adult zebrafish	*Lactobacillus plantarum*	Direct inoculation in rearing water	Decrease in anxiety like behavior, increase in *gabra1* and *slc6a4a* in the brain.	Davis et al. (2016b)
Adult zebrafish	*Lactobacillus delbrueckii*	Direct inoculation in rearing water	Decrease in anxiety like behavior and increase in expression of *gad1* in brain and gut.	Olorocisimo et al. (2023)
Adult zebrafish	*Paraburkholderia sabiae*	Direct inoculation in rearing water	Decrease in anxiety like behavior, increase in exploratory behavior, increase in levels of taurine in brain and gut, increase in expression of *oxti*, *bdnf*, and *tph2* in brain, no change in expression levels of GABAergic genes and decrease in abundance of *Actinobacteria* and *Chloroflexi* in treatment group.	Ichikawa et al. (2023)
Adult zebrafish	Melatonin	Direct inoculation in rearing water	Alteration in levels of γ-GABA, dopamine and serotonin, and alteration in intestinal microbiota where microbiota community structure in melatonin and probiotic treatment group was comparable to control group, change in intestinal metabolism as well as increase in acetic and propionic acid following melatonin treatment in a neurotransmitter secretion disorder model.	Zhang et al. (2021b)
	*Lactobacillus plantarum* HNU082			
Adult zebrafish	Amoxicillin	Direct administration in rearing water	Decrease in diversity of microbial community in the intestine, altered behavior showing anxiety like behavior, and increase in corticotrophin releasing hormone.	Deprey (2016)
Adult zebrafish	Amoxicillin	Direct administration in rearing water	Exposure to high dose (100 mg/L) of Amoxicillin decreased social interaction behavior, locomotor activity, catalase activity and increase in superoxide dismutase activity in the brain tissue.	Gonçalves et al. (2020)
Larval zebrafish	*Aeromonas veronii*	Immersion	Increase in locomotor activity in germ free larvae and colonization with *Aeromonas veronii* or *Vibrio cholerae* blocked the hyperactive locomotion and distinct microbial communities among germ free, germ free colonized and conventionally colonized larval zebrafish.	Phelps et al. (2017)
	*Vibrio cholerae*			
Larval zebrafish	Metabolites from zebrafish intestine	Immersion	Delay in expression of *notch1b*, *ngn1*, *ascl1a*, decrease in expression of *fgf8* and *phox2bb* which is involved in neurodevelopmental in germ free compared to conventionally raised and treatment with zebrafish metabolites in germ free larvae rescued the expression of the neural development genes.	Rea et al. (2022)
Adult zebrafish	Morphine	Direct administration with feed in rearing water	Administration of Sinomenine inhibited the dependence on morphine in zebrafish, diminished the changes in bacterial community in gut, restored the reduced expression levels of *occludin a* and *b* in the brain and inhibited expression of *il1b* in brain and intestine in morphine treatment group.	Chen et al. (2020)
	Sinomenine			
Immune				
Germ free larvae	*Aeromonas*	Immersion	Colonization of germ-free zebrafish with zebrafish derived bacterial isolates, *Vibrio* showed significant increase in the number of neutrophils response while *Aeromonas* showed no clear relationship between neutrophil response and bacterial abundance when compared to germ free showing that immunostimulatory effects varies among bacterial species.	Rolig et al. (2015)
	*Vibrio*			
	Shewanella			
	Variovorax			
	Delftia			
	*Acinetobacter*			
	*Aeromonas* sp. 2			
	*P*seudomonas			
	*Pleisomonas*			
	*Enterobacter*			
Embryos	Lipopolysaccharide (LPS)	Immersion	Embryos exposed to LPS induced activation of pro-inflammatory cytokines such as TNF-α and IL-1β.	Watzke et al. (2007)
Germ free larvae	*Aeromonas veronii* biovar sobria	Immersion	Mono-colonization of germ-free larvae with gram negative bacteria (*Aeromonas* and *Pseudomonas* species) induced intestinal alkaline phosphatase (AP) activity comparable to conventionalized larvae while mono-colonization with gram positive bacteria isolates (*Streptococcus* and *Staphylococcus* species) showed no induction of intestinal AP activity. Exposure to LPS showed reduced expression of TNF-α and TNF-β in myd88-MO compared to wild-type. Authors also showed that germ free larvae had no neutrophils in the intestine compared to wild type.	Bates et al. (2007)
	*Pseudomonas fluorescens*			
	*Streptococcus*			
	*Staphylococcus*			
	LPS			
Adult zebrafish	Adherent-invasive *Escherichia coli* (AIEC)	Bath inoculation	Infection with AIEC upregulated intestinal expression of pro-inflammatory cytokines such as TNF-α, IL-1β, IFNγ and S100A-10b (like human calprotectin). Treatment with probiotic EcN rescued the pro-inflammatory response, tissue damage and decreased AIEC colonization.	Nag et al. (2022)
	*E. coli* Nissle 1917 (EcN)			
Immune—Dietary Factors				
Larval zebrafish	Butyrate	Immersion	Exposure to butyrate, but not acetate or propionate decreased recruitment of neutrophils following a tail wound injury comparable to levels seen with dexamethasone treatment. Butyrate also reduced macrophage and TNF-α recruitment at the wound site while propionate increased macrophage recruitment.	Cholan et al. (2020)
	Acetate			
	Propionate			
	Dexamethasone			
Larval zebrafish	Acetic acid	Immersion	Treatment with TNBS induced colitis with increased tissue damage, inflammation, gut dysbiosis as seen with increase in *Betaproteobacteria* and *Actinobacteria*, decreased goblet cells and endocytic function. Treatment with acetic, propionic, and butyric acid (SCFAs) reduced expression of inflammatory cytokines (*il1b* and *il6*), recruitment of neutrophils in the intestinal, maintained bacterial population level in gut comparable to control, but did not restore tissue damage in intestinal wall or number of goblet cells.	Morales Fénero et al. (2021)
	Propionic acid			
	Butyric acid			
	2,4,6-trinitrobenzene sulfonic acid (TNBS)			
Larval zebrafish	High fat diet (control diet with 10% w/w of cocoa butter)	Direct administration with feed in rearing water	Administration of high fat diet for 25 days in larval zebrafish induced microbial dysbiosis with increase in the abundance of *Bacteroidetes*, upregulation in the expression of inflammatory cytokines (*IL22*, *IL1B*, *TNF*, *MYD88*, *NFκB*), receptors involved in host-microbiome interaction (TLR2, TLR5, NOD1), antimicrobial peptides (IAP, MPO, MMP9, DEF1), damaged intestinal barrier and increased goblet cell production in comparison to control group on a normal diet.	Arias-Jayo et al. (2018b)
Larval zebrafish	Soybean-meal based diet	Direct administration with feed in rearing water	Soybean meal diet induced a Th17 response in zebrafish compared to control diet. Administration of soybean-based meal diet in a Rag1 deficient larvae produced no inflammation response showing a T-cell dependent inflammatory response.	Coronado et al. (2019)
Adult zebrafish	Sea buckthorn polysaccharide	Direct administration with feed in rearing water	Supplementation of sea buckthorn polysaccharide with high fat diet improved the survival rate significantly with a decrease in levels of triglycerides, total cholesterol, aspartate aminotransferase, alanine transaminase in the serum, lipid accumulation in the liver, increased expression of *Nrf2*, *Cu/Zn-SOD*, and *TGF-β1* in the intestine and liver. Dietary supplementation of sea buckthorn also corrected the dysbiosis in microbial community by promoting proliferation of beneficial intestinal bacteria (e.g.,: *Cetobacterium*) compared to zebrafish on high fat diet alone.	Lan et al. (2022)
Adult zebrafish	High fat diet	Direct administration with feed in rearing water	Supplementation of a probiotic like protein (AM-ZHY1) with Amuc-100 anchored to surface of *Lactobacillus lactis* ZHY1 with high fat diet showed a significant decrease in hepatic steatosis, decreased expression of *PPARy*, *SREBP-1c*, *FAS*, *ACC1*, *CD36*, *FABP6*, *TNF-α*, and *IL6* in the liver, increased expression of intestinal tight junction proteins and reduced abundance of *Proteobacteria* and *Fusobacteria* compared to high fat diet alone.	Zhang et al. (2021a)
	Amuc_1100,			
	*Lactobacillus lactis* ZHY1			
Adult zebrafish	Exopolysaccharide (EPS) from *Lactobacillus rhamnosus* GG (LGG) and *L. casei* (BL23)	Direct administration with feed in rearing water	Administration of EPS derived from LGG promoted microbial homeostasis, activates HIF1α in the intestine which increases expression of antimicrobial peptides and has decreased expression of pro-inflammatory cytokines in the liver while EPS derived from BL23 induced liver inflammation with an increase in ALT, AST levels in the serum, increased expression of *TNF-α*, *IL-6*, *IL-10* and gut microbial dysbiosis. EPS from both strains reduced hepatic steatosis in zebrafish on high fat diet and increased levels of intestinal acetate and propionate compared with high fat diet.	Zhang et al. (2019b)
Adult zebrafish	*Lactobacillus rhamnosus* GCC-3 EPS	Direct administration with feed in rearing water	Supplementation of diet with EPS from GCC-3 improved the survival rate of zebrafish infected with spring viremia of carp virus compared to control. There was also an increased expression of type I interferon and improved the gut microbiota homeostasis. *In-vitro* studies with fibroblast cells from zebrafish showed that culture of supernatant of GCC-3 EPS associated microbiota inhibited replication of the spring viremia of carp virus compared to culture supernatants on control diet.	Xie et al. (2022)
Larval zebrafish	High fat diet (emulsion of chicken egg yolk)	Direct administration in egg water	High fat diet inhibited nutrient sensing, decreased EECs signaling activity, altered the microbial composition in the gut and changed morphology of the EECs to a more “close type” that lacks apical extension increased abundance of *Acinetobacter* bacteria compared to control group.	Ye et al. (2019)
Juvenile zebrafish	Butyrate	Direct administration with feed in rearing water	Butyrate and saponin supplemented feed increased expression of pro-inflammatory genes and activity of oxidoreductase, decreased expression genes involved in histone modification, mitotic process, GPCR activity, and increased relative abundance of *Rhodobacter*, *Flavobacterium* and *Bacteroides,* which are associated with inflammation in juvenile zebrafish gut, increased eosinophils, rodlet cells and reduced mucus producing cells in the gut compared to control diet.	López Nadal et al. (2023)
	Saponin			
Immune—Environmental Factors				
Adult zebrafish	Perfluoroalkyl phosphonic acids	Direct exposure in rearing water	Exposure to perfluoroalkyl phosphonic acids induced anxiety like behavior, increased abundance of gram-negative bacteria leading to increased levels of LPS in the brain, serum, and gut, increased expression of *iL-1B* and decreased expression of *iL-10* in the brain, compared to control. Increased levels of LPS in the brain also altered permeability of the blood-brain barrier as seen by a decrease in the expression of tight junction proteins, leading to brain injury as seen by damaged myelin sheath fibers, increase in ROS production and apoptotic cells in the brain. Co-exposure of perfluoroalkyl phosphonic acid with CH (AhR inhibitor) decreased expression of *ARNT1*, *CYP1A1* and *iL-1B* and increased expression of *iL-10* in the brain while also decreasing anxiety like behavior relative to treatment of perfluoroalkyl itself.	Zhang et al. (2023)
	Antibiotics			
	AhR inhibitor			
Adult zebrafish	Triclosan,	Triclosan was directly administered in rearing water	Supplementation with *Lactobacillus plantarum* diminished toxic effects due to triclosan exposure, restored levels of triglycerides and total cholesterol, decreased number of CD4 T cells in the lamina propria of duodenal mucosa, and decrease in expression of intestinal pro-inflammatory cytokines (NF-κB, and lysozyme). Supplementation with *Lactobacillus plantarum* also decreased anxiety like behavior and improved learning/memory following chronic Triclosan exposure.	Zang et al. (2019)
	*Lactobacillus plantarum* ST-III	Lyophilized bacteria supplemented in feed		
Adult zebrafish	Microcystin-LR (MC-LR)	Direct exposure in rearing water	Exposure to MC-LR or/and GLY decreased expression of *claudin-5*, *occludin*, *zonula occludens-1*, increased intestinal permeability, increased levels of intestinal IL-1β and IL-8, *p53*, *bax*, *bcl-2*, *caspase-3*, *caspase-9*, activated activity of superoxide dismutase, increased relative abundance of *Fusobacteria* and *Proteobacteria*. PCRUSt analysis showed alteration in pathways involving lipid metabolism, circulatory metabolism, energy metabolism, metabolism of cofactors, vitamins, replication and repair, and translation.	Ding et al. (2021)
	Glyphosate (GLY)			
Adult zebrafish	Polystyrene microplastics	Direct exposure in rearing water	Exposure to 100nm and 200 µm of polystyrene microplastics caused an increased in levels of TNF-a and TLR 2 in the intestine, increased abundance of pathogenic bacteria while the lowest concentration (100 nm) also induced increased expression of genes related to ROS production and increased production of mucus secreting cells. Exposure to the polystyrene microplastics also increased the abundance of pathogenic bacteria such as *Actinobacillus*, *Mycoplasma* (100 nm), *Staphylococcus* (5 µm), *Vibrio*, *Acinetobacter*, *Porphyromonas*, *Haemophilus*, *Neisseria*, *Lactococcus* (200 µm) in the intestine of zebrafish.	Gu et al. (2020)
Adult zebrafish	*Microcystis aeruginosa*	Direct inoculation in rearing water	Exposure to *M. aeruginosa* increased number of intestinal goblet cells, inflammation, and relative abundance of *Cyanobacteria*, *Actinobacteria* at the phylum level and abundance of *Vibrio*, *Lactobacillus*, *Shewanella*, *Methylobacterium*, *Pseudomonas* and *Halomonas* at the genus level.	Qian et al. (2019)

## Utilizing zebrafish as a model to explore host-microbiota interactions in cardiovascular health and disease

The cardiovascular system in the zebrafish is comprised of a single closed circulatory system, with two chambers and four components that include sinus venous, atrium, ventricle and bulbous arteriosus (Gut et al., 2017; Galanternik et al., 2020). The heart is also one of the first organs to form during the early developmental stages in organogenesis (Stainier, 2001) and it is assumed to have its adult morphology by 5 dpf (Stainier and Fishman, 1994). Despite some anatomical differences between the mammalian and zebrafish heart (e.g., four versus two chambers), there exists a level of conservation both anatomically and physiologically. For example, the zebrafish heart rate averages 120–180 beats per minute (bpm), closer to humans (60–100 bpm) (Poon and Brand, 2013), compared to other widely used animal models such as mice (at 550 to 620 bpm) (Kass et al., 1998). Due to this, the zebrafish is highly utilized in cardiovascular and metabolic research including in research of atherosclerosis, cardiomyopathies, congenital heart disease, fatty liver disease, obesity, and diabetic retinopathy, reviewed in detail by Gut et al. (2017). Remarkably, zebrafish can survive without a beating heart for up to 7 dpf, and their ability to utilize passive gas exchange by diffusion across body surface makes them invaluable in research of hemodynamic forces in cardiac development (Andrés-Delgado and Mercader, 2016) and reperfusion injury (Zou et al., 2019). Adult zebrafish heart can regenerate following mechanical injury or genetic manipulations, which makes it an attractive model of cardiac injury such as myocardial infarction (Poss et al., 2002; Wang et al., 2011; Gut et al., 2017).

At 24 hpf, the vascular cord develops into the dorsal aorta and the cardinal vein, marking the beginning of blood circulation and angiogenesis (Gut et al., 2017). Just like the heart, the zebrafish vascular system is readily accessible to study blood and lymphatic vessel formation, owing to their optical clarity that enables single cell resolution in the larvae or the transparent zebrafish models (Stainier and Fishman, 1994). In addition to the transgenic models with manipulated vascular function, techniques like microangiography (McKinney and Weinstein, 2008), combined with high power microscopy techniques such as differential interference microscopy, selective plane illumination microscopy (Hoffmann et al., 2022), confocal microscopy (Ye et al., 2019; Ye et al., 2021), light sheet microscopy (Taormina et al., 2012), two-photon microscopy (Renninger and Orger, 2013; de Vito et al., 2022) and three-photon microscopy (Hontani et al., 2022), allow for *in vivo* real time measurements in this model. Physiologic measurements of blood pressure, although difficult, are also possible using the servo-null micro-pressure system in zebrafish larvae (Pelster and Burggren, 1996; Kopp et al., 2005), rendering the zebrafish a highly valuable model in the study of cardiovascular health (Bakkers, 2011). Other techniques employed to study the overall function of the zebrafish heart include measurements of blood flow and velocity (Shin et al., 2010; Jamison et al., 2013), heartbeat measurements using noninvasive (Chan et al., 2009) and semi-automated techniques (Fink et al., 2009), and measurements of stroke volume, ejection fraction, and cardiac output (Parker et al., 2014). Parker et al. developed a larval zebrafish model that can simultaneously report chronotropic, inotropic, and arrhythmic effects, as well as blood flow and vessel diameter (Yalcin et al., 2017). While challenging, Salehin et al. (Salehin et al., 2021) demonstrated a technique to measure intraventricular pressure in 3-5 dpf larval zebrafish, while Hu et al. (2001) was able to record pressure gradients from atrium to the ventricle and from ventral to dorsal aorta using similar techniques in the adult zebrafish (3–6 months). As a surrogate to direct blood pressure measurements, Schwerte et al. utilized digital motion analysis tools to measure distribution of red blood cells in the peripheral vascular system of larval zebrafish (Schwerte and Pelster, 2000).

The renin-angiotensin system (RAS) is a hallmark of cardiovascular disease and plays a crucial role in regulation of blood pressure, fluid and electrolyte balance, and vascular tone in health and disease (Levy, 2005; Parker et al., 2014). In addition, RAS has been implicated in modulation of the gut bacteria in mammalian models (Wang et al., 2015; Santisteban et al., 2017; Donertas Ayaz et al., 2021; O’Donnell et al., 2023); however, equivalent studies of RAS-microbiota interactions in zebrafish are limited. This is despite the fact that the components of RAS are relatively conserved across vertebrates, and zebrafish transgenic lines have been generated to explore this system (Liang et al., 2004; Hoffmann et al., 2022) using imaging and various molecular techniques (Taormina et al., 2012; Quan et al., 2020; Salehin et al., 2021). Zebrafish have at least one ortholog of the key mammalian RAS components such as *ace* and *ace2* (Postlethwait et al., 2021), and Fimasartan, an angiotensin II receptor antagonist, has been shown to exert protective effects in heart failure in zebrafish (Quan et al., 2020). Another study by Rider et al. showed that expression of renin was modulated following inhibition of angiotensin converting enzyme using Captopril and altered salinity, but showed no change following renal flow ablation in larval zebrafish (Rider et al., 2015). The availability of transgenic lines and mutant zebrafish described in (Rider et al., 2015; Hoffmann et al., 2022) enables the use of zebrafish to study microbiota-RAS interactions.

In addition to hypertension, dyslipidemia and atherosclerosis are risk factors for cardiovascular diseases. Studies show that gut microbiota influence lipid levels in the host by producing secondary bile acids with effects on the hepatic and systemic lipid metabolism as well as glucose metabolism (Tang et al., 2017), while atherosclerotic plaques can contain intestinal bacterial DNA (Tang et al., 2017), and administration of genetically engineered probiotic species can modulate glucose in mammalian models (Verma et al., 2019). Zebrafish are frequently utilized in similar studies. A review by Vasyutina et al. (Vasyutina et al., 2022) highlights the use of zebrafish as a model to study dyslipidemia and atherosclerosis, due to the ease of genetic manipulation, high throughput genetic screening, conservation among lipid metabolism pathways and homology of genes such as *apob*, *apoe*, *apoa1*, *apoc2*, *ldlr*, *lpl*, and *cetp* in the zebrafish. Furthermore, Ka et al. (2020) showed an evolved convergent mechanism for regulating lipid metabolism in the zebrafish on high fat diet via transcriptomic profiling. Another study utilized microbiome-depleted larval zebrafish to perform transplantation of microbiota from mouse on high fat diet to study effect of mammalian high fat diet on hyperlipidemia (Manuneedhi Cholan et al., 2022). They showed that larval susceptibility to hyperlipidemia was driven by *Stenotrophomonas maltophilia* and *Enterococcus faecalis,* as evidenced by the increase in lipid droplet accumulation in larvae on a chicken egg yolk diet, while inoculation of *E. faecalis* in zebrafish larvae with *myd88* knockdown showed significantly less lipid droplet accumulation (Manuneedhi Cholan et al., 2022). Furthermore, administration of *Lactobacillus rhamnosus* increased the relative abundance of *Firmicutes,* decreased total body cholesterol and triglyceride content, lipid droplet size in the intestine and increased the length of microvilli and enterocytes (Falcinelli et al., 2015). Another study showed that administration of *Lactobacillus rhamnosus* in zebrafish larvae showed a decrease in orexigenic genes and increase in anorexigenic genes, reducing glucose levels and appetite (Falcinelli et al., 2016). A study by Willms et al. utilized intestinal cell transcriptomics to demonstrate a decrease in pro-angiogenic factors in germ-free larval zebrafish (Willms et al., 2022), suggesting microbes may play a role in intestinal angiogenesis. Moreover, modelling poor dietary practices in zebrafish by overfeeding and exposure to diethylhexyl phthalate, an obesogenic pollutant present in plastics, demonstrated alterations in the gut microbiota and their metabolism of carbohydrates, fatty acids, and lipids (Buerger et al., 2020), similar to what had previously been reported in rodent models (Hur and Lee, 2015; Boulangé et al., 2016; Tilg et al., 2020). In view of this, studies targeting the modulation of gut microbiota by bacterial colonization either in larval (Arias-Jayo et al., 2018a; Ye et al., 2019) or adult zebrafish (Nag et al., 2018), and those investigating the role of major bacterial metabolites such as short chain fatty acids (SCFAs) (Poll et al., 2020) in cardiovascular health in zebrafish are warranted. SCFAs have been shown to modulate blood pressure, lipid metabolism, and glucose metabolism in the host (Pluznick, 2013; Koh et al., 2016; Lu et al., 2022). However, some questions remain, such as the ability of the zebrafish gut bacteria to produce SCFAs, or whether these would have any effects on cardiovascular and metabolic parameters in the fish as in mammalian models. Cholan et al. showed that microbiota of adult zebrafish can produce three major SCFAs (acetate, propionate, and butyrate) *in vitro*; however, the *in vivo* detection of fecal SCFAs was unsuccessful, possibly due to detection limitations (Cholan et al., 2020). To our knowledge, there are no studies investigating the effects of SCFAs on cardiovascular parameters using zebrafish. In this regard, the high throughput capacity of this model can be utilized in combination with microscopy, bacterial colonization, and metagenomics in transgenic lines to identify gut bacteria and their metabolic byproducts that can serve as biomarkers in cardiovascular health and other conditions.

## Zebrafish as a model to explore the microbiota-neural axis

Like the mammalian nervous system, the nervous system in the zebrafish has a central- (CNS) and peripheral arm (PNS). The development of the zebrafish brain and the CNS occurs within 3 dpf (d’Amora and Giordani, 2018). Additionally, the ENS is the largest subset of the PNS that innervates the GI tract that originates from the vagal neuronal crest (Kaslin et al., 2020). The vagus nerve is a component of the PNS and a major mediator of gut-brain neural interactions in maintenance of host homeostasis (Ma et al., 2019) whose signaling can be modulated by the gut microbiota (Breit et al., 2018), begins forming at 3 dpf in the zebrafish (Olsson et al., 2008). A recent study by Ye et al. performed live calcium imaging of intestinal EECs in free-swimming zebrafish larvae following exposure to *E. tarda*. They elegantly showed that vagal innervation in the zebrafish extends to the GI tract, where the vagus forms a direct contact with a subpopulation of intestinal EECs (Ye et al., 2021). Similarly, enteric innervation in the mid and distal intestine is observed by 3 dpf, and in the proximal intestine at around 12 dpf (Olsson et al., 2008).

The overall developmental plan of the nervous system is relatively conserved between species in morphology and composition (Kaslin et al., 2020). Not only do the zebrafish have similar brain morphology to mammals, with the CNS regions such as the telencephalon, diencephalon (forebrain), mesencephalon (midbrain), metencephalon (hindbrain), and spinal cord that are formed around 1 dpf (Kaslin et al., 2020), they also exhibit conserved neurochemistry, with the formation of neuronal subtypes such as dopaminergic and oxytocinergic neurons completed at 2 dpf (Machluf et al., 2011). In line with this, a study by Higashijima et al. demonstrated that a transgenic zebrafish line can be used to characterize cranial nerves in the larvae (Higashijima et al., 2000), while external fertilization of zebrafish embryos also enable *in vivo* study of neural patterning and neuronal specification (Machluf et al., 2011). Zebrafish also possess a functioning blood brain barrier that develops in early larval stages and can be utilized in studies of permeability and transport (Jeong et al., 2008; Eliceiri et al., 2011).

The CNS, via PNS, is known to interact with the gut and plays a role in intestinal motility, secretion, absorption, immune function, and overall intestinal homeostasis (Rhee et al., 2009; Carabotti et al., 2015). The sensory communication between the gut and the brain is mediated via the vagal and spinal sensory nerve afferents (Carabotti et al., 2015; Yu et al., 2020), while the nervous system can, in turn, modulate the GI environment and function via motor nerves and direct hormonal effects (Berthoud, 2008; Browning and Travagli, 2014; Carabotti et al., 2015; Fung et al., 2019; Menon et al., 2019). Bacteria can produce major neurotransmitters like serotonin (Carabotti et al., 2015; Fung et al., 2019; Chen et al., 2021), modulate the production of host neurotransmitters (Appleton, 2018) and neurotrophic factors (Maqsood and Stone, 2016), in part via gut bacterial metabolites like SCFAs (Vecsey et al., 2007; Ryan et al., 2016; Silva et al., 2020). In addition, the gut microbiota is reportedly involved in regulating neurogenesis, maturation of microglial cells, regulation of stress responses and the hypothalamic-pituitary axis, as well as in maintenance of the blood brain barrier integrity (Dash et al., 2022). Thus, it is of no surprise that an imbalance in the abundance or composition of gut bacteria has recently been shown to increase the risk of neuropsychiatric disorders like anxiety, depression, Parkinson’s disease (Finegold et al., 2002; Bale et al., 2010; Al-Asmakh et al., 2012; Dash et al., 2022), schizophrenia (Finegold et al., 2002; Bale et al., 2010; Al-Asmakh et al., 2012; Dash et al., 2022), and in neurodevelopmental disorders such as autism spectrum disorder (Wang et al., 2012) and attention deficit hyperactivity disorder (Iliodromiti et al., 2023).

Owing to the similarities between the mammalian and zebrafish gut and nervous system function as well as the microbiota composition, zebrafish are an attractive model to study host-microbiota interactions in neural health. For example, the effects of the gut microbiota on stress behaviors using germ-free larval (Davis et al., 2016a) and adult zebrafish (Davis et al., 2016b) have been established. In these studies, the absence of endogenous microbiome promoted anxiety-like behaviors and perturbed GABAergic and serotonergic signaling pathways, like what has been reported in mammalian models (Martin et al., 2009; Olivier et al., 2013). These behavioral deficits in the zebrafish were alleviated by administration of *Lactobacillus plantarum* in rearing water (Davis et al., 2016a; Davis et al., 2016b), suggesting a modulating role for bacteria in zebrafish behavior. Along the same lines, microbiota in adult zebrafish can be manipulated by long-term administration of lyophilized probiotics (Borrelli et al., 2016). In one study, administration of *Lactobacillus rhanmosus* modified shoaling behavior and expression of several genes involved in neural signaling and metabolism of major neurotransmitters, suggesting the probiotic effects on the zebrafish brain (Borrelli et al., 2016). More recently, administration of *Lactobacillus delbrueckii* by direct inoculation into fish tanks resulted in reduced anxiety-like behavior in adult female zebrafish, altering its gut and brain transcriptomes (Olorocisimo et al., 2023). A similar study by Ichikawa et al. showed that administration of *Paraburkholderia sabiae* in rearing water reduced anxiety-like behaviors and increased diversity of gut microbiota in adult zebrafish (Ichikawa et al., 2023). In investigation of neural hyperactivity, supplementation with *Lactobacillus plantarum* HNU082 in the feed influenced select neurotransmitters in adult zebrafish (Zhang et al., 2021b). In contrast, Tian et al. showed that administration of typical fluoroquinolone antibiotics (Enrofloxacin) via incubation induced anxiety-like behaviors and gut dysbiosis demonstrated by increased *Bacteroidetes* and a decrease in the F/B ratio in the gut of adult zebrafish (Tian et al., 2023). Similarly, decreased social interaction and altered locomotion was observed following administration of Amoxicillin, a broad spectrum antibiotic previously associated with decreased richness in gut bacteria, in zebrafish (Deprey, 2016; Gonçalves et al., 2020). Zebrafish have also been used to model an intricate neuropsychiatric illness schizophrenia by studying shoaling behavior (Ewald, 2009), which can also be affected by the gut bacteria (Borrelli et al., 2016).

The microbiome can also shape the development of the zebrafish CNS. For example, Phelps et al. demonstrated that gut microbiota plays a key role in zebrafish neural development (Phelps et al., 2017). The authors employed a standard locomotor assay using conventionally colonized, germ-free and germ-free colonized larval zebrafish via incubation with *Aeromonas veronii* or *Vibrio cholerae*. The study revealed that germ-free larval zebrafish had an increase in locomotor activity compared to the colonized groups, suggesting that presence of the microbiota in early stages of life is critical in neurological development of zebrafish (Phelps et al., 2017), similarly to what has been shown in mammals (Laue et al., 2022). Other studies that employed whole mount *in situ* hybridization and gene ontology analyses in germ-free zebrafish larvae and those treated with gut-derived metabolites (Rea et al., 2022) showed that alterations in a key neurodevelopmental signaling pathway can be attributed to the effects of gut microbiota. Another intriguing study showed that morphine dependence can be modified by antibiotic treatment in the adult male zebrafish (Chen et al., 2020), suggesting a potential for zebrafish in study of host-microbiota interactions in drug abuse. A study by Demin et al. (2018) discussed the use of zebrafish to study the endocannabinoid and opioid system as a new target for therapeutics. Such zebrafish models can also be utilized to explore the role of the gut microbiota, considering its association with anxiety (Lee et al., 2021), pain (Minerbi and Shen, 2022), and drug use (Volpe et al., 2014; Chen et al., 2020; Rueda-Ruzafa et al., 2020). Thus, elucidating gut microbiota-host interactions in the zebrafish model may open new therapeutic possibilities for behavioral disorders (Li et al., 2021; Samochowiec and Misiak, 2021) in the identification and functional characterization of bacterial biomarkers for future diagnostics. In addition, zebrafish can serve as a useful tool to bridge the gap in our understanding of the interaction between gut bacteria and xenobiotics in host neurotoxicity, as discussed by Bertotto et al. (2020) especially since the use of zebrafish is well established in neurotoxicology studies with standardized behavior and molecular assays.

The ENS, a component of the autonomic nervous system, is crucial for intestinal motor function, mucosal transport and secretion, modulation of immune and endocrine activity, and maintenance of the healthy gut microbiota in the zebrafish (Costa et al., 2000; James et al., 2019; Hamilton et al., 2022). One of the most common ENS dysfunction is Hirschsprung disease, a congenital condition that is commonly characterized by absence of enteric neural crest cells in the distal region of the GI tract (Kuil et al., 2020). This absence can be due to defects in proliferation, migration, and differentiation of enteric neural crest-derived cells (Mueller and Goldstein, 2022). A study by Rolig et al. showed that zebrafish lacking the ENS due to a mutation in *sox10*, a known HSCR gene, showed a delay in intestinal transit, increased levels of neutrophils in the intestine, increased abundances of pro-inflammatory bacterial communities, and a decrease in the abundance of *Firmicutes*, *Bacteroidetes*, and antimicrobial peptides (Rolig et al., 2017). Numerous other studies also show that specific gut microbiota can shape the intestinal environment, gut neurotransmitter secretion, and inflammatory responses in the zebrafish (Jeong et al., 2008; Eliceiri et al., 2011; Roach et al., 2013; James et al., 2019; Ye et al., 2019; Hamilton et al., 2022) similar to what is reported in mammalian models (Gomaa, 2020; Chen et al., 2021; de Vos et al., 2022). A landmark study utilized state of the art *in vivo* imaging to demonstrate a role for specific gut bacteria via tryptophan metabolites in modulation of the GI vagal sensory signaling to the hindbrain in zebrafish larvae (Ye et al., 2021), opening a world of possibilities for the use of this model to study the effect of gut microbiota on the gut-brain vagal axis. All these studies highlight a role for the gut microbiota in mediating gut-brain communication, neural function and development, neural disorders, and metabolism of neuromodulating drugs in the zebrafish model, thus underscoring the potential of the zebrafish model to elucidate specific mechanisms involved in both microbial and host-related processes.

## Utilizing the zebrafish microbiome as a functional biomarker of the host immune responses

The immune system plays a key role in sustaining a healthy balance between host and the invading pathogens including viruses and bacteria (Wu and Wu, 2012; Childs et al., 2019). However, exaggerated immune responses are deleterious in many conditions. The cause and effect of these responses remain largely unknown, and the complexity of immune interactions justifies the demand for animal models. Evaluation of the immune system function in the zebrafish is justified by significant similarities to the mammalian system: i) the presence of both innate and adaptive immune systems; and ii) the presence of two primary lymphoid organs; a kidney marrow, which is similar to bone marrow in mammals, and the thymus and spleen as secondary lymphoid organs (Wattrus and Zon, 2018). Hematopoiesis also occurs similarly to mammals, with generation of primitive red cells occurring at around 26 hpf in the zebrafish (Traver et al., 2020). The primitive immune cells such as myeloid cells give rise to macrophages and neutrophils, first in the yolk sac around 24 hpf, then in the mesenchyme of the head and in the circulation (Herbomel et al., 1999). Lymphoid immune responses in zebrafish are relatively slower compared to those in mammals, as B cells and antigen-binding B cells are not observed until 7–10 dpf and 10–14 dpf respectively (Traver et al., 2020). The zebrafish innate immune system acts as the first line of defense, similar to mammals, showing phagocytic activity following microbial infections as early as 1 dpf (van der Vaart et al., 2012). Due to its transparent body at early stages of development, the zebrafish model is useful to study immune interactions in real time using imaging techniques such as differential interference contrast microscopy (Herbomel et al., 1999; Herbomel and Levraud, 2005) or by utilizing transgenic zebrafish lines with fluorescently labelled immune cells (Torraca et al., 2014). Torraca et al. discuss how bacterial and fungal infections can be modeled in zebrafish embryos to study immune defense mechanisms (Torraca et al., 2014), with the availability of transgenic zebrafish lines allowing in depth exploration of the immune system at a cellular level (Traver et al., 2020). Zebrafish also undergo antibody recombination similarly to mammals (Traver et al., 2020), and they possess several conserved mammalian orthologs of Toll-like receptor (TLR), which are extensively studied for their ability to recognize pathogens as the first line of defense (van der Vaart et al., 2012). Moreover, exposure of zebrafish to pathogenic bacteria results in elevation of activating transcription factors (ATF), nuclear factor-κβ (NFκβ), Ap-1, interferon regulatory factors (IRF), and STAT transcription factor families (Stockhammer et al., 2009), reflecting another similarity to mammalian responses. However, there are also dissimilarities, as the zebrafish encode fish-specific innate immune response receptors such as the Novel Immune-Type Receptor family, Diverse Immunoglobulin Domain-Containing Protein Family, which are the zebrafish natural killer-like cells, Polymeric Immunoglobulin Receptor-Like Family, and Leukocyte Immune-Type Receptors (Traver et al., 2020). In addition, zebrafish lack lymph nodes (Miao et al., 2021), and possess three classes of immunoglobulins (IgD, IgM, and IgZ) in contrast to the five classes seen in mammals (IgA, IgD, IgE, IgG and IgM) (Traver et al., 2020; Miao et al., 2021).

As in mammals, there is evidence for the role of the gut microbiome in development of the zebrafish immune system (Masud et al., 2017). For example, Rolig et al. (2015) studied bacterial-bacterial interactions by measuring neutrophil responses in germ-free zebrafish larvae compared to those mono-colonized by select gut bacteria. A study by López Nadal et al. (2020) demonstrated a bidirectional interaction between the zebrafish immune system and its microbiota, as transgenic zebrafish lacking macrophages showed significant gut dysbiosis with a reduction in relative abundances of *Fusobacteria*, α- and γ-*Proteobacteria*, and an increase in *δ-Proteobacteria*. In support of the symbiotic relationship, Galindo-Villegas et al. showed that germ-free zebrafish larvae, via TLR/MyD88 signaling, produced dampened proinflammatory responses to viral infections compared to the conventionally raised larvae, suggesting a role for commensal microbiota in mediating zebrafish immune responses to infection (Galindo-Villegas et al., 2012) similarly to what is observed in mammals (Wirusanti et al., 2022). Bates et al. also showed that lipopolysaccharide, a component of the outer membrane of gram-negative bacteria and a pro-inflammatory mediator, can promote GI epithelial cell differentiation in the zebrafish (Bates et al., 2006b) and increase the expression of pro-inflammatory factors like TNF-α and IL-1β in zebrafish embryos (Watzke et al., 2007), similarly to mammals (Barko et al., 2018). In germ-free zebrafish, however, exposure to lipopolysaccharides also reportedly produced an anti-inflammatory response involving *iap*/TLR/MyD88 to modulate TNF signaling and maintain host homeostasis (Bates et al., 2007; Koch et al., 2018), suggesting species differences in some responses.

Reciprocally, zebrafish immunity can alter the composition of the gut microbiota. For instance, compared to the wild-type zebrafish, mutants lacking the *rag1* gene exhibit elevated levels of pathogenic *Vibrio*, while re-introduction of T lymphocytes from conventionally-raised zebrafish via adoptive transfer decreased the abundance of *Vibrio* in *rag1*-deficient zebrafish (Brugman et al., 2014). Zebrafish models have also been utilized to study the effects of human intestinal pathogens such as the adherent-invasive *Escherichia coli* (AIEC), associated with Chron’s Disease and ulcerative colitis (Nag et al., 2022). For example, bath inoculation with AIEC colonized the adult wild-type zebrafish causing upregulation of inflammatory markers such as IL-1β, TNF-α, IFN-γ, and S100A-10b, while co-inoculation with the probiotic *E. coli* Nissle decreased colonization of AIEC and reduced inflammatory responses (Nag et al., 2022). Thus, the zebrafish presents an especially useful model to study host immune system-microbiota interactions, while the use of microbiota as a biomarker of host immune dysfunction may be particularly attractive.

### Diet and the microbiota-immune axis in zebrafish

One of the key exogenous factors in regulation of host-microbiota interactions is diet. One major marker of dietary influences on the microbiota is the production of SCFAs, byproducts of fermentation of dietary fiber (Nogal et al., 2021). In addition, composition of microbiota is different at different developmental stages (5,10, 35, 70 dpf) and diets, including at individual bacterial taxa level (Wong et al., 2015). In mammalian species, SCFAs exert anti-inflammatory effects, and can enhance the gut barrier function and modify metabolic function by providing sustenance to the GI epithelium (Kolodziejczyk et al., 2019). Cholan et al. (2020) also demonstrates the anti-inflammatory properties of butyrate in the zebrafish larvae, as exposure to butyrate via immersion reduced neutrophil migration and M1-type proinflammatory macrophages following a tail wound injury. This was consistent with another study that found that pre-treatment with butyrate increased survival and decreased production of TNF-α following exposure to LPS in 3 dpf larvae (Wang, 2023). Noteworthy, a recent high throughput quantitative histological and transcriptomic analyses of the zebrafish gut showed that sodium butyrate supplemented in feed increased the expression of pro-inflammatory genes and activity of oxidoreductase, decreased expression of genes involved in histone modification, mitotic process, GPCR activity, and increased relative abundance of *Rhodobacter*, *Flavobacterium* and *Bacteroides,* which are associated with inflammation in juvenile zebrafish gut (López Nadal et al., 2023). Histological analysis also showed that butyrate supplementation increased eosinophils, rodlet cells and reduced mucus producing cells in the gut (López Nadal et al., 2023), suggesting the responsiveness of the zebrafish gut immune system to the changes in the gut microbiota. Moreover, Morales Fenero et al. showed that SCFA can increase survival rate, reduce expression of inflammatory cytokines, and reduce the recruitment of neutrophils in a colitis model induced by 2,4,6-trinitrobenzene sulfonic acid in larval zebrafish (Morales Fénero et al., 2021). While treatment with SCFA did not restore the presence Goblet cells, it did maintain intestinal function and normalized the abundance of *Betaproteobacteria* and *Actinobacteria* (Morales Fénero et al., 2021). A high fat diet, a known pro-inflammatory and pro-dysbiosis modulator in mammalian models (Cani and Delzenne, 2009; Hersoug et al., 2016; Malesza et al., 2021) can also alter the gut microbiome composition, damage intestinal barrier, and elevate NF-κβ-mediated systemic inflammation in zebrafish (Arias-Jayo et al., 2018b). In addition, an *in vivo* measurements of EEC activity demonstrated a role for high fat in inhibition of nutrient sensing and disrupted the morphology of EECs while causing gut dysbiosis in the zebrafish (Ye et al., 2019). Coronado et al. showed that administration of inflammatory diet (soybean meal-based diet) stimulated a Th17 response in zebrafish models (Coronado et al., 2019). Furthermore, the same inflammatory diet produced no inflammation in *rag1*-deficient zebrafish, suggesting that diet-induced inflammation was T cell-dependent in the zebrafish (Coronado et al., 2019). Another study by Lan et al. (2022) showed that dietary supplementation of sea buckthorn polysaccharide in male wild-type zebrafish on a high fat diet reduced liver and intestinal inflammation while promoting the growth of beneficial bacteria in the zebrafish intestine.

Colonization of the host gut with bacteria is a useful tool to assess causation. However, while immersion of the zebrafish has been shown to be successful in the larvae, alternative techniques are necessary for successful transplant of anaerobic gut bacteria in the fish. Considering that repeated oral gavage may be technically challenging and stressful for the model, administration of lyophilized bacterial strains in adult zebrafish on high-fat diet has been utilized to modify the endogenous gut microbiota and the host immune system and metabolism (Liu et al., 2023). For example, high fat diet supplemented with Amuc_1100, a protein derived from the probiotic *Akkermansia muciniphila,* can modify endogenous gut microbiota and reduce host weight and inflammation, while improving the intestinal barrier in the adult zebrafish (Zhang et al., 2021a). Another study utilized dietary delivery of lyophilized exopolysaccharides extracted from *Lactobacillus rhamnosus* to demonstrate its protective role in the adult zebrafish (Zhang et al., 2019b; Xie et al., 2022). As manipulation of the gut bacteria reportedly produces similar immune responses in zebrafish to those observed in mammalian models, further investigation of the host-microbiota interactions as a marker of the immune health in zebrafish is warranted.

### Environmental contaminants and microbiota-immune axis in zebrafish

Long-term exposures to pollutants such as nano- and microplastics can alter the characteristics of the microbiota and lead to immune and other disorders (Hirt and Body-Malapel, 2020; Zhang et al., 2022). Similarly, this can also affect wildlife health (Fackelmann and Sommer, 2019), and gut microbiota are a potential tool for detection of compromised aquatic conditions (Abdul Razak and Scribner, 2020; Kim et al., 2021; Turner et al., 2022). However, a direct link between pollutants, microbiota and health in the aquatic species is limited due to lack of established controlled environments. One recent study showed that exposure to polystyrene microplastics altered gene expression of pro-inflammatory cytokines such as TNF-α and TLR-2 in adult zebrafish (Gu et al., 2020). This study utilized single cell RNA sequencing of the zebrafish intestine following polystyrene microplastic exposure in a population of 12,000 intestinal cells to identify the diverse gene expression profiles. They showed that polystyrene microplastic induced immune cell dysfunction, having the most significant effect on secretory cells of the GI tract (Gu et al., 2020). This was associated with increased abundances of pathogenic bacteria, and altered functionality of select immune cells in the zebrafish intestine (Gu et al., 2020). More recently, study by Zhang et al. (2023) showed that exposure to perfluoroalkyl phosphonic acids (commonly found in aquatic environments) induced anxiety-like behaviors and increased lipopolysaccharides, resulting in intestinal inflammation and blood-brain barrier disruption in adult male zebrafish. In another study, chronic exposure to triclosan, an antimicrobial compound and an environmental pollutant commonly found in medical and chemical applications, altered the gut microbiota and lipid metabolism, increased production of inflammatory cytokines, and modified the behavior of exposed zebrafish (Zang et al., 2019). This was counteracted by supplementation with *Lactobacillus plantarum* ST-III, which reduced the presence of inflammatory cells and markers in the intestine, and rebalanced gut bacterial communities in the adult zebrafish (Zang et al., 2019). Another study showed that exposure to triclosan in larval zebrafish increased abundance of genera *Rheinheimera* in 10 dpf larvae (Koch et al., 2018). Phylogenetic analysis predicted upregulated microbial pathways including antibiotic resistance, sulfonation, drug metabolism and oxidative stress, suggesting that gut microbes may play a role in the biotransformation of triclosan (Weitekamp et al., 2019). These studies signify a functional role for the microbiota in modulation of pollutant effects on the immune system in aquatic species, beyond its potential use as a biomarker of aquatic conditions.

Zebrafish are also emerging as an important model for studying the pathophysiological effects of toxins produced during harmful algal blooms on aquatic wildlife. Microcystin is a hepatotoxin produced by *Microcystis* and *Planktothrix*, two genera of cyanobacteria that dominate freshwater harmful algal blooms, for example, in Lake Erie (U.S.A) and Lake Taihu (China). In larval zebrafish, microcystin activated endoplasmic reticulum stress, lowered the heart rate, and triggered heart muscle cell apoptosis (Qi et al., 2016). In adult zebrafish, microcystin affected reproduction by reducing spawning activity and potentially preventing the release of eggs (Baganz et al., 1998). Most alarmingly, zebrafish models have demonstrated parental transmission of microcystin toxicity. When parents were exposed to microcystin, their unexposed F1 offspring exhibited lower hatching success, decreased body length and weight, and suppression of immune genes IFN-1 and TNF-α compared to offspring of unexposed parents (Liu et al., 2014). Moreover, the antioxidant system in zebrafish offspring was destroyed by microcystin exposure in the parental generation, as indicated by occurrence of lipid peroxidation in the F1 offspring (Liu et al., 2014). Furthermore, exposure of adult zebrafish to *Microcystis aeuroginosa*, a primary species of toxin-producing cyanobacteria, (Qian et al., 2019), or Microcystine-LR and glyphospate, the latter a common carcinogen found in aquatic environments, (Ding et al., 2021), altered the abundance of gut microbes and induced intestinal inflammation. Considering these and other studies, continued improvements in methodology to investigate host-microbiota interactions in the zebrafish are justified in mammalian and aquatic research.

## Summary and future directions

While the gut microbiota composition can vary between species and conditions, significant overlap between the zebrafish and mammalian gut microbiota exists, and the similarities in physiologic responses to the gut bacteria in the zebrafish and mammalian models justify its increasing use in modeling of host-microbiota interactions in health and disease. The use of zebrafish in high throughput studies can provide a deeper insight on the intricate crosstalk between the gut microbiota and the host systems. This can lead to better understanding of the pathophysiology of human conditions such as hypertension, cardiovascular diseases, neurological disorders, immune dysfunction, and discoveries of more effective therapeutic targets. Future studies should utilize genome-editing tools combined with high resolution microscopy to explore the interactions between microbiota and the complex host multi-systems at a molecular and single cell level *in vivo*.
